# Affective Response During a Sorting Task for Geriatric Hoarding

**DOI:** 10.1093/geroni/igaa057.972

**Published:** 2020-12-16

**Authors:** Mary Dozier, Amy Young, Scott Camilleri

**Affiliations:** 1 Mississippi State University, Mississippi State, Mississippi, United States; 2 Mississippi State University, Mississippi State, United States

## Abstract

Previous research suggests older adults with hoarding disorder are more likely to express non-fear-based emotions when sorting. The purpose of this study was to examine the emotions expressed by eight rural-dwelling older adults with hoarding disorder when sorting and discarding possessions. The study took place in participants’ homes and involved a 15-minute behavioral sorting task where participants were asked to sort through personal items and make a decision to either keep or discard each item. Participants were asked to rate their Subjective Units of Distress (SUDs) and to state their emotional state prior to the sorting task, every 5 minutes throughout the test, and after completing the task. Four participants did not complete the sorting task due to a lack of a desire to discard objects (e.g., “I want to keep it all”). For the four participants who did complete the task, an average of 53 items were sorted and an average of 24 items were discarded. The average decrease in SUDs from pre-task to post-task was 40. Overall, most participants reported feeling positive emotions before, during, and following the task, with the most commonly reported emotion being joy. This study supports prior research suggesting that not all hoarding is fear based and that older adults may be more motivated by increasing positive associations with sorting and discarding. A focus on increasing patients’ insight and sense of self-efficacy may lead to increased treatment gains for older adults with hoarding disorder.

